# Involvement of SPATA31 copy number variable genes in human lifespan

**DOI:** 10.18632/aging.101421

**Published:** 2018-04-19

**Authors:** Cemalettin Bekpen, Chen Xie, Almut Nebel, Diethard Tautz

**Affiliations:** 1 Max-Planck Institute for Evolutionary Biology, 24306 Plön, Germany; 2 Institute of Clinical Molecular Biology, Kiel University, 24105 Kiel, Germany

**Keywords:** SPATA31, primary foreskin fibroblast, senescence, human lifespan

## Abstract

The *SPATA31 (*alias *FAM75A)* gene family belongs to the core duplicon families that are thought to have contributed significantly to hominoid evolution. It is also among the gene families with the strongest signal of positive selection in hominoids. It has acquired new protein domains in the primate lineage and a previous study has suggested that the gene family has expanded its function into UV response and DNA repair. Here we show that over-expression of *SPATA31A1* in fibroblast cells leads to premature senescence due to interference with aging-related transcription pathways. We show that there are considerable copy number differences for this gene family in human populations and we ask whether this could influence mutation rates and longevity in humans. We find no evidence for an influence on germline mutation rates, but an analysis of long-lived individuals (> 96 years) shows that they carry significantly fewer *SPATA31* copies in their genomes than younger individuals in a control group. We propose that the evolution of *SPATA31* copy number is an example for antagonistic pleiotropy by providing a fitness benefit during the reproductive phase of life, but negatively influencing the overall life span.

## Introduction

Expansion of gene families with the concomitant acquisition of new functions can be a driving force for the evolutionary differentiation of species. Compared to other mammals, primate and human genomes include many interspersed segmental duplications, which may have been of special relevance for the evolution of the primate lineage [1]. These segmental duplications range between one to several hundred kilobases, and are characterized by a mosaic of repeat structures. They can be associated with rapid structural changes and chromosomal instability. About 430 blocks of the human genome have been identified as having been subject to multiple duplications during hominoid evolution [1]. Clustering analysis of these segmentally duplicated regions in the human genome suggests that a part of the duplication blocks have formed around a “core” or “seed” duplicon [2,3]. Some of the most variable human CNV genes correspond to recently evolved gene families among the human core duplicons (e.g. *NPIP* and *LRRC37A*) [4].

The *SPATA31* gene family belongs to the core duplicon gene families and it has been shown to be one of the fastest evolving gene families in the human lineage [5]. We have previously characterized in detail the structure, transcriptional pattern, and protein localization of the *SPATA31* gene family [6]. It has expanded from a single copy in mouse to at least nine copies in humans, located at seven different sites on both arms of chromosome 9. The coding regions are part of larger segmental duplications and one can distinguish two types, *SPATA31A* and *SPATA31C*. Type A is annotated with seven segmental duplications in the human reference genome (genome build hg38), including two pseudogenes, type C is annotated with two copies. The gene lengths and the protein coding regions of *SPATA31* genes differ between A and C types, but not much within each type. Compared to the mouse gene, we found that the human *SPATA31* genes are broadly expressed and have acquired new functional domains, among them a cryptochrome/photolyase domain, suggesting the acquisition of a function in UV damage repair. Antibody staining showed that the protein is re-localized from the nucleolus to the whole nucleus upon UV irradiation, a pattern known for proteins involved in UV damage sensing and repair. Based on CRISPR/Cas mediated knockouts of members of the gene family in fibroblast cell cultures, we found that the reduction of copy number in cells leads to enhanced sensitivity towards UV-irradiation. Given that increased UV-light resistance of the skin may have played a major role in human evolution, we proposed that the acquisition of an involvement in UV damage sensing or repair has lead to the adaptive evolution of *SPATA31* [6].

An interesting side effect of the *SPATA31* gene knockouts was that the respective cells survived somewhat longer than normal primary fibroblast cell lines, although this was difficult to quantify. We have therefore used here the alternative approach, namely to over-express a representative member of the *SPATA31* gene family, *SPATA31A1,* and study its effect on cell survival. We find that this over-expression results indeed in premature senescence of the cells, through interference with known aging related pathways. Based on these results, we asked whether natural copy number variation in humans correlates with senescence, in the sense that fewer *SPATA31* copies should correlate with longer life span. We can indeed show this effect in a cohort of long-lived individuals. Humans that have reached an age of 95 or higher have on average fewer *SPATA31* gene copies than a younger control population.

## RESULTS

**Figure 1:**
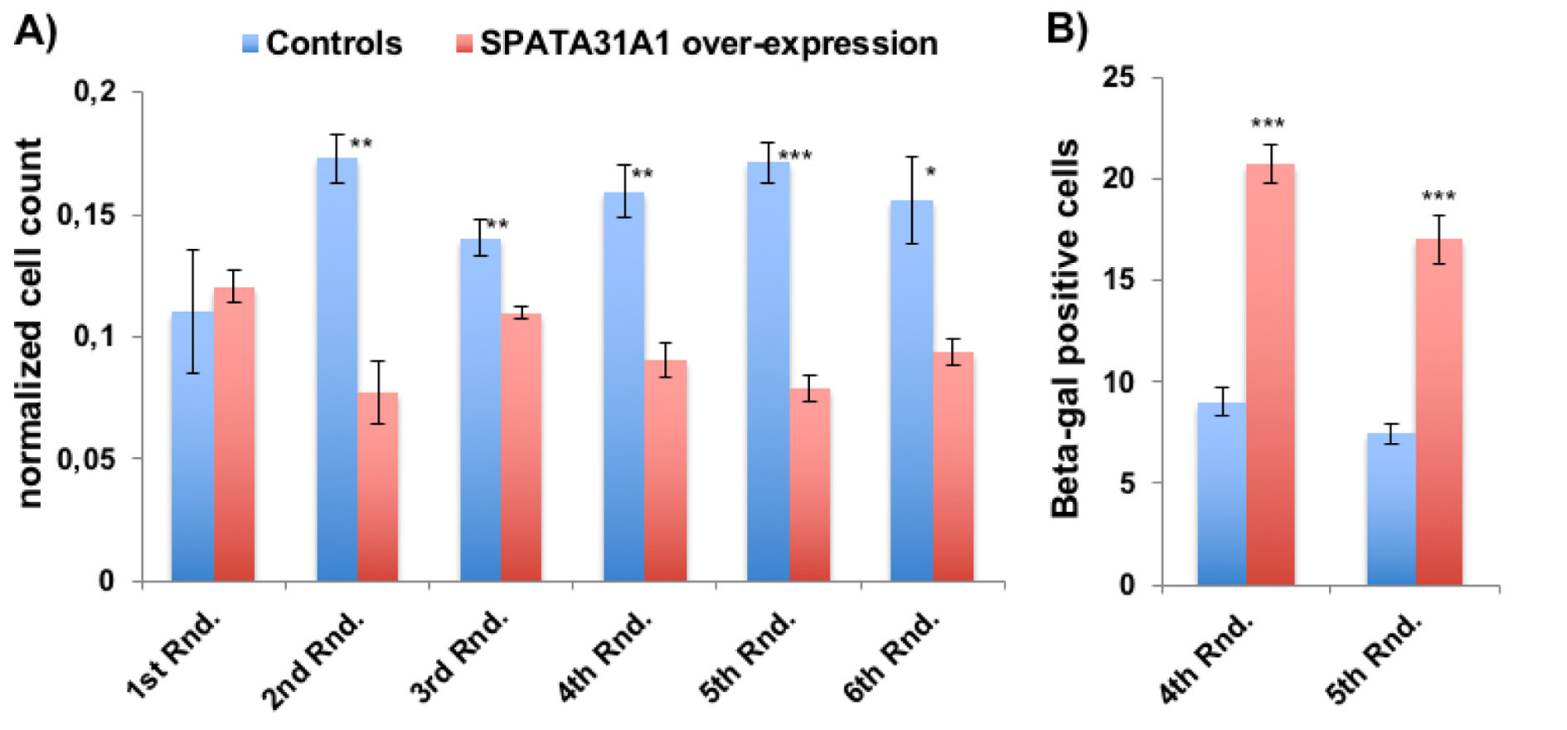
**SPATA31A1 over-expression leads to reduced cell growth and increased senescence.** (**A**) Relative number of cells per flask after each round of 3 weeks of culturing. The first round (1^st^ Rnd) represents the normalized cell numbers three weeks after the initial seeding. Differences in normalized cell numbers became significant after the 2nd round, up to the 6th round when the experiment ended. The blue bars represent the averages and standard error of the four replicates of the control cells and the red bars the SPATA31A1 over-expressing cells. The numbers were normalized with respect to total cell counts. See Suppl. Figure S1 for the immunofluorescence images of control-eGFP and SPATA31A1-eGFP expression. (**B**) Senescence of SPATA31A1 over-expressing cells detected by the ß-galactosidase assay. The fraction of ß- galactosidase (+) staining cells from the 4^th^ and 5^th^ Rnd of re-culturing are shown with standard error between replicates. See suppl. Figure S2 for an example of staining results for senescence-associated ß-galactosidase assay. P-values indicated above the bars (*p< 0.05, **p< 0.01, ***p< 0.001; student t-test).

Over-expression of *SPATA31A1* in primary human fibroblast (HFF) cells was achieved using a mammalian expression vector with a CMV enhancer and promoter. Normal expression of *SPATA31A1* is low in these cells, while the introduction of the expression vector resulted in a 2.7 fold increase. After the initial transformation, cells were re-cultured every three weeks for up to five additional rounds. We found that SPATA31A1 over-expressing cultures produced relatively fewer cells in each of the replication rounds than the controls transformed with the vector only (Fig. 1A). Based on the ß-galactosidase staining assay for cellular senescence, we observed about twice as many senescent cells in the *SPATA31A1* over-expressing cultures than in the controls (Fig. 1B).

RNA sequencing of the cells transfected with only the vector (control) or the *SPATA31A1* expression plasmid (treatment) after one week of culture (i.e. before differences in cell numbers would become apparent) was used to study the influence of *SPATA31A1* on expression networks. Of 23,723 expressed genes in these cells, 8.5% (2,023) showed significant up- or down-regulation (5% false discovery rate) (Suppl. Table S1a,b; Suppl. Figure S3). Functional gene enrichment analysis [7] (Table 1, Suppl. Table S2) was applied to assess the affected pathways. The three most significant KEGG pathways that changed upon over-expression of *SPATA31A1*, namely cell cycle [8], PI3K-Akt signaling pathway [9] and extracellular matrix (ECM) receptor interaction [10] are known to play a role in aging. ECM homeostasis is centrally involved in UV-damage repair of fibroblasts [11], confirming the previous notion that *SPATA31* gene functions are part of this pathway [6]. ECM receptor interaction was also previously shown to be differentially regulated between senescent and young cells [12].

**Table 1:** Pathway enrichment analysis.

**KEGG Pathway Term**^1^	**ID**	**Input number**	**Background number**	**P-Value**	**Corrected P-Value**
Cell cycle	hsa04110	39	124	1.16e-11	3.29e-09
Pathways in cancer	hsa05200	73	399	2.50e-10	3.54e-08
ECM-receptor interaction	hsa04512	27	83	9.08e-09	6.95e-07
Proteoglycans in cancer	hsa05205	45	208	9.82e-09	6.95e-07
PI3K-Akt signaling pathway	hsa04151	61	343	1.83e-08	9.33e-07

^1^The corresponding analysis was performed using the web server (http://kobas.cbi.pku.edu.cn/) [7]. Only the five most significant KEGG pathways are listed (full list in Suppl. Table S2).

Among 66 genes that showed a more than 2-fold change (Suppl. Table S1b), we found eleven that had previously been noticed to be differentially regulated or involved in aging or age-related diseases (up-regulated: *BEX1* [13], *SERPINB2* [13], *CORIN* [13], *CHI3L1* [14], *STMN2* (*SCG10*) [15]^,^ [16] and [13], and *PTGS2* (*COX2*) [17,18]; down-regulated: *VANGL2* [13], *CYTL1* [13], *IGF2* [19] and *CDH 6* [20]) (Table 2). *IGF1*, which has been implicated in aging and UV-damage repair [21], is also significantly down-regulated, but less than 2-fold (Table 2).

**Table 2:** Expression changes for genes discussed in the text.

**Gene ID**	**Ensembl ID**	**baseMean^1^**	**log2FoldChange^2^**	**adjusted p**
*BDNF*	ENSG00000176697	3401	-0.31	1.31E-06
*BEX1*	ENSG00000133169	166	2.07	1.53E-40
*CDH6*	ENSG00000113361	1514	-1.02	1.91E-44
*CHI3L1*	ENSG00000133048	112	2.11	4.40E-51
*CORIN*	ENSG00000145244	218	1.45	3.05E-38
*CYTL1*	ENSG00000170891	26	-1.00	2.73E-08
*DTL*	ENSG00000143476	258	0.77	3.91E-10
*IGF1*	ENSG00000017427	58	-0.62	1.02E-03
*IGF2*	ENSG00000167244	10998	-1.02	2.28E-38
*PCNA*	ENSG00000132646	2062	0.32	1.31E-05
*PTGS2*	ENSG00000073756	1120	1.59	1.45E-62
*SERPINB2*	ENSG00000197632	120	1.55	1.22E-30
*SPATA31A1*	ENSG00000204849	19	1.45	6.48E-18
*STMN2*	ENSG00000104435	806	1.65	1.78E-74
*VANGL2*	ENSG00000162738	131	-1.12	1.75E-16

^1^baseMean represents the normalized read counts from all samples

^2^log2FoldChange is the calculated fold expression change based on normalized read counts, (full list in Suppl. Table S1b).

We asked further whether other differentially expressed genes are known to be associated with aging effects. We found that 21% (57 out of 278 expressed in the HFF cells) of age-related genes in the GenAge Database [22] overlap with our list of differentially expressed genes, including *PCNA*, *BDNF*, *IGF1* and *IGF2* (Table 2 and Suppl. Table S1c). Hence, the expression data corroborate the observation of increased senescence of the *SPATA31A1* over-expressing cells in culture.

*SPATA31A* genes carry a PCNA (proliferating cell nuclear antigen) interacting domain [6] and *PCNA* is significantly up-regulated in *SPATA31A1* over-expressing cells (Table 2). PCNA is a cofactor of DNA polymerase delta and was found to be one of the genes involved in translesion DNA synthesis and repair [23]. Therefore, we focused on additional DNA repair genes by extracting the respective GO terms from the Gene Ontology database (Amigo2 accession GO:0006281). Twelve percent (57 out of 472 expressed in the HFF cells) of these DNA repair genes are significantly differentially expressed in our data (44 up and 13 down; Suppl. Table S1d). Some of the up-regulated genes are directly related to PCNA dependent translesion repair genes. This includes DTL (Cdt2-CRL4 complex) which is involved in PCNA-dependent translesion DNA synthesis [24,25].

**Figure 2:**
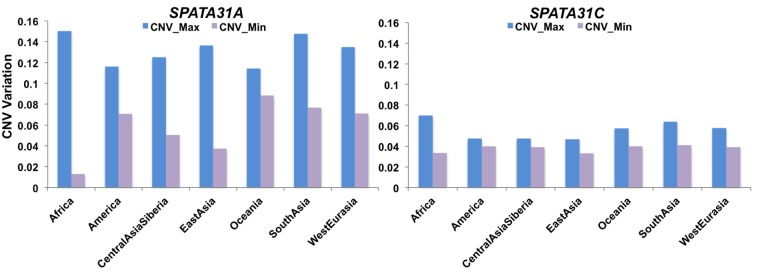
**Range of *SPATA31* copy numbers in different human population groups.** The data are based on normalized read depth from the Simons Genome Project data [26] from which we have also retrieved the classification into population groups. The Y-axis represents normalized read depth measures, minimum and maximum values are provided for each group.

These results raise the question whether natural expression differences of *SPATA31* genes through copy number variation could influence mutational and aging processes in humans, especially since humans have the relatively highest number of *SPATA31* gene copies among primates [6]. To obtain an overall pattern of copy number variation in humans, we analyzed the Simons Genome Project (SDGP) [26] data using a read depth approach. Given the differences between the SPATA31 A- and C-types (see introduction), we analyzed them separately. We found that *SPATA31A* has more copies and is more copy number variable than *SPATA31C* (Fig. 2), with the largest copy number differences seen for *SPATA31A* in the African populations. When summed across all *SPATA31* copies, averages and variances are similar between the population groups, with the exception of native Americans and Africans having lower averages and Africans also higher variance (Suppl. Figure S4). Given that higher copy numbers are expected to express also more gene product, we use in the following correlations with copy numbers to assess effects of *SPATA31* in humans.

First we asked whether *SPATA31* gene copy number could differentially affect the germline mutation rate, especially since *SPATA31* is highly expressed in testis [6]. We have tested this by analyzing the data from 250 parent-offspring trios from the Genome of the Netherlands **(**GoNL) consortium re-sequencing data [27]. We extracted *SPATA31* copy numbers of the parents from the genome sequence data and compared these with the numbers of germline mutations detected in this project. We did not find a significant correlation between *SPATA31* copy numbers of either parent with *de novo* mutations in their children (Suppl. Figure S5; Suppl. Table S3), suggesting that the SPATA31 function in the germline does not have an effect on the mutation rate. However, it seems possible that the germline function is anyway different from its somatic function (see Discussion).

**Figure 3:**
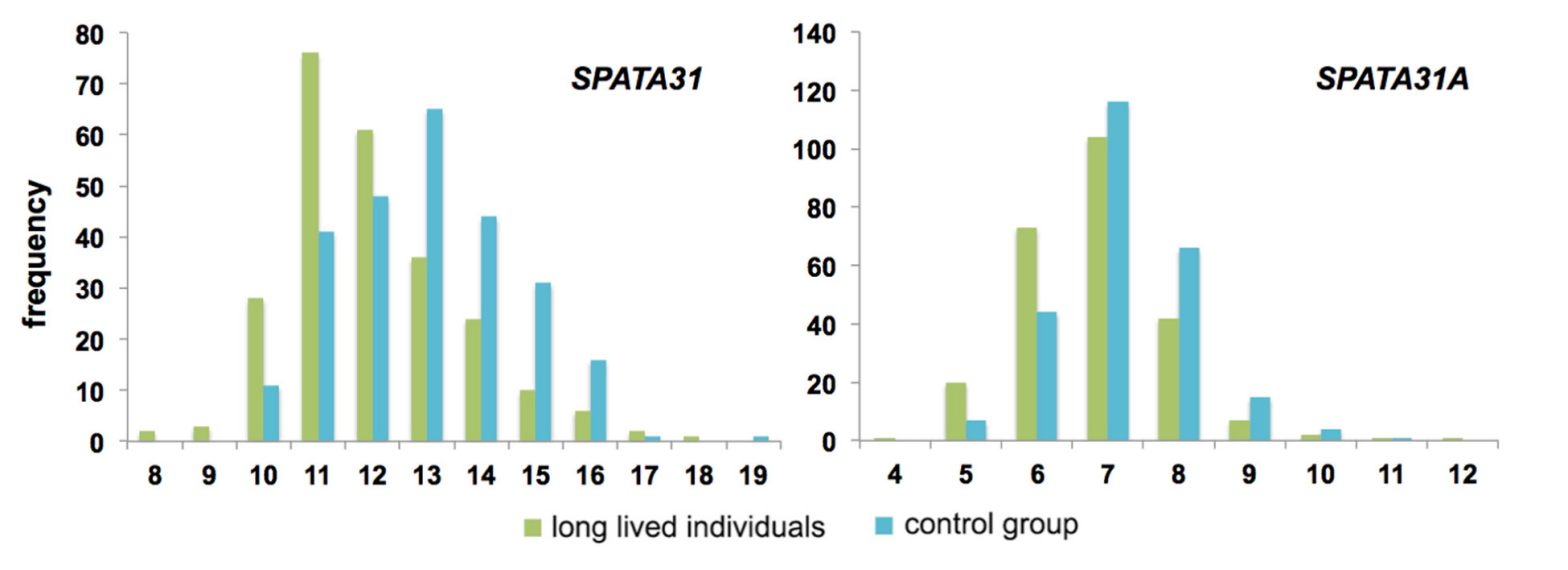
**Distribution of copy number classes of *SPATA31* genes between long-lived individuals (n=258) and controls (n=249).** Long-lived individuals were older than 96 years and control individuals between 60-75 years at the time of sampling [28,29]. Copy number variations were determined by digital PCR using primers that amplify all possible *SPATA31* copies (left) or only SPATA31A copies (right). The distributions are significantly different (both p<< 0.0001, Wilcoxon rank sum test).

There are currently no datasets that would allow testing whether natural somatic mutation rates correlate with *SPATA31* copy numbers. But the observation of an increased senescence phenotype in cells with over-expression of *SPATA31A1,* as well as the expression changes in genes related to aging, prompted us to ask whether copy number variation in human populations could be related to longevity in humans. We analyzed a human DNA sample collection of long-lived individuals (LLI) more than 96 years old and compared it to an average population control of individuals aged 60 to 75 years [28,29]. Since most of the individuals in the control group are still alive, they may include also individuals that become very old. However, by statistical criteria, fewer than 2% are expected to become centenarians [29]. Quantitative droplet PCR was used to measure *SPATA31* copy numbers in these samples. We found that LLI had indeed on average a significantly lower copy number than the control group (Fig. 3; Suppl. Table S4).

Given the differences in variability between the *SPATA31A* and *SPATA31C* copies, we designed also primers specific for the *SPATA31A* variants and retested the LLI and control panels. We found the same general pattern as for the full gene set, with LLI having on average significantly fewer copies of *SPATA31A* (Fig. 3; Suppl. Table S4).

## DISCUSSION

The segmentally duplicated genes in primates and humans have received special attention, since they may have significantly contributed to the evolution of these species [1]. However, only few of these genes have been studied in functional detail so far. Together with our previous analysis of the evolution of the *SPATA31* gene family [6], the present data suggest that *SPATA31* genes are involved in sensing and repairing UV-induced DNA damage, but also in the induction of pathways causing the premature aging of cells. Further, we show that long lived individuals have on average lower numbers of *SPATA31* genes in their genome.

The ancestral function of the gene has been in spermatogenesis. Knockouts for *SPATA31* in mice lead to spermatogenesis defects and infertility [30]. Given that *SPATA31* genes are also highly expressed in the testis of humans, it seems possible that they still have the same function in humans. This would explain why we find no effect on the germline mutation rate, since the testis function would be different from the function in the rest of the body. The specialization for sensing UV-damage and its repair has apparently evolved only together with the acquisition of the new protein domains in primates [6]. The increase in copy number may then have become beneficial as a response to an increased exposure to UV light as consequence of a switch from nocturnal to diurnal lifestyle, as well as increasing loss of body hair in humans. But our results from the over-expression of SPATA31A in epithelial cells suggest that this comes with a cost, namely an increased activation of senescence pathways, coupled with an enhancement of DNA repair processes, which could lead to more somatic mutations.

It has generally been suggested that there is a complex interaction between cellular senescence, tumor incidence due to somatic mutations and aging [31]. Our data imply that *SPATA31* genes are part of this process and that their variation in copy number contributes via this effect to longevity in humans. Having more copies may lead to more somatic mutations, including some that cause cancer, while having fewer copies reduces this effect, thus allowing longer life spans. But given that also other senescence pathways are activated by over-expressing *SPATA31*, the link to life span may also be more complex.

The *SPATA31* copy number effect on aging can be seen as an example for antagonistic pleiotropy [32,33]. Higher copy numbers provide a benefit early in life, due to better protection of the skin against sun light, allowing to spend more time during the day for foraging, social life, mate seeking and child care, all factors that should increase reproductive fitness. Hence, there would be positive selection for higher copy numbers. But more copies would also lead to a higher expression of SPATA31 and our cell-culture results show that such a higher expression induces DNA repair pathways. This could lead to a higher incidence of repair-induced damage in the cells and thus to cancer. If this becomes a problem during reproductive age, one would have a potential negative selection against high copy number. Hence, a balance in copy number should be maintained in the population, but with a certain variance. This variance has the effect that total lifetime beyond reproductive age is affected, with individuals with fewer copies having a higher probability to live longer.

Although somatic mutations in humans have long been known to depend on UV-light exposure among other factors [34], the molecular pathways remain to be studied further. *SPATA31* copy number variation could turn out to be a risk factor for an increased somatic mutation rate, in particular in combination with UV-exposure [35]. We have not studied this particular aspect here, but it seems possible that humans with a higher sensitivity towards sunlight may have on average fewer *SPATA31* copies, implying at the same time that they could have a longer life span.

There have been a series of SNP based genome-wide association studies (GWAS) in humans to detect loci associated human longevity (e.g [29,33,36–38]. and references therein). Several associations were also detected on chromosome 9 (http://genomics.senescence.info/longevity/ [37], which harbors the SPATA31 copies, but none of these regions is close to the location of the mapped functional copies of SPATA31 along the chromosome. In fact, it is unlikely that causative copy number polymorphisms would be picked up in GWAS experiments, since copy number changes tend to evolve faster than their associated SNPs. There have also been two association studies for mortality in long-lived individuals which have focused on copy number variants [39,40]. However, the regions identified in these studies do not overlap with the annotations of the *SPATA31* genes. One possible reason may be that the *SPATA31* copies occur at multiple places along chromosome 9 and the effect on aging may not be related to any one particular of them. Only screens that would take interaction effects into account might be able to uncover them.

It is currently difficult to estimate how fast copy numbers can change between generations and over time, since this would require phasing of the clusters in family studies, which is currently technically too challenging. We can currently also not rule out the possibility that there is somatic variation in SPATA31 copy number, since we do not have a direct comparison to germline derived copy numbers. Hence, also the modes of inheritance of this copy number variation, for example whether new copy number variation arises frequently between generations or even somatically, remains a question for the future.

## METHODS

### Over-expression of *SPATA31A1*

*SPATA31A1* constitutes a representative member of the *SPATA31A* genes [6]. A cDNA was obtained from GE Healthcare-Dharmacon (MGC cDNA Clone ID:9056783, MHS6278-213246307) and cloned N-terminally into the mammalian expression vector pEGFPN1 in frame by using EcoR1 (5’) and Kpn1 (3’) restriction enzymes (NEB) (Suppl. Figure S6). Clones were confirmed for integrity by Sanger sequencing. DNA was prepared by using the EndoFree Plasmid Maxi Kit (Qiagen Cat No:12362). Human Foreskin Fibroblast cells (HFF) were purchased from the American Type Culture Collection (ATCC) (HFF2703, CRL-2703). Two rounds of four independent transfections for each of the pSPATA31A1-EGFP (pS) and vector controls pEGFP (pE) were performed using Amaxa Basic Nucleofector Kit Primary Mammalian Epithelial Cell (Lonza Cat No: VPI-1005). Transfected cells were grown in IMDM growth medium (Gibco Cat no: 21980-065) supplemented with 10% FBS and G418 antibiotic (200ng/mL) to ensure that only successfully transfected cells would grow. Suppl. Figure S1 provides examples for the immunofluorescence analysis of the transfected cells. Cells were incubated at 37°C in 5% CO_2_ atmosphere as a pH regulator. Once the cells reached a sufficient density for splitting, 50,000 cells of each transfection were sub-cultured into 75mm flasks. After 3 weeks of culture, cells were treated with 0.05% Trypsin (Gibco Cat No: 25300-054) to dissociate them from the flask. Equal amount of medium was added and cells were pelleted by centrifugation for 5 min at 300g. Cells were re-suspended in 4 mL of fresh medium and counted with a hematocytometer. Counting was performed always in pairs of each control and treatment group to keep the cells under the same conditions. 50,000 cells were then transferred into a new flask and remaining cells were frozen for long-term preservation. This procedure was repeated every three weeks until the 6^th^ round.

### Cellular senescence assay

Senescence associated ß-galactosidase expression was assayed using the ß-gal senescence kit from Sigma (Cat No: CS0030-1KT). The test was performed by seeding 50,000 cells from each culture in the 4^th^ and the 5^th^ round of culturing (84^th^ day and 105^th^ day) on 35mm dishes. After 3 days of growth, cells were analyzed according to the manufacturer’s instruction. To obtain better staining, the staining solution was replaced after 4 hours of initial incubation and then cells were incubated overnight at 37°C. After completion of staining (Suppl. Figure S2 provides examples of stained cells), 10 randomly selected fields were counted from each dish, using a Leica inverted microscope (20x objective), The fraction of stained cells was counted in each field amounting to a total count of about 250 cells per dish.

### RNASeq analysis

50,000 cells from preselected culturing at round 0 (before the 1^st^ round) were transferred into 5 cm dishes for each culture. HFF cells were then incubated for an additional week in IMDM growth medium supplemented with 10% FBS and 200ng/ml G418 as selection agent. Total RNA was isolated using the RNeasy Mini Kit (Qiagen Cat No: 74104) and subjected to high throughput sequencing as paired end reads with 2 x 150 cycles (Illumina NextSeq 500). Libraries were prepared with the TruSeq stranded mRNA Library Prep Kit.

The sequencing reads were first quality checked using FastQC (https://www.bioinformatics.babraham.ac.uk/projects/fastqc/) and trimmed using Trimmomatic [41]. This yielded on average 30 million paired-end reads that were mapped to the human genome hg38 with tophat2 [42] and read counts per gene annotated in ENSEMBL hg38 were retrieved by featureCounts [43]. The DESeq2 software package 1.8.2 [44] was used to detect significant differential expression between genes of pE1-pE4 cells (control) and pS1-pS4 cells (treatment). Suppl. Table S5 provides the read statistics.

### Analysis of GoNL data

We retrieved genome read data for 250 offspring trios (in total 750 individual bam files) with permission of the Genome of Netherlands Project (GoNL) application number 2016126 approved by GoNL Data Access Committee on 12-05-2016 [27]. Total mapped reads of *SPATA31* and single copy reference house keeping genes (*UBE2*, *mTOR*, *TBP*, *B2M* and *TERT*) (see Suppl. Figure S7 for coordinates) were extracted using samtools. The resulting total reads were mapped against the human reference genome (build hg38) using Bowtie2 [45]. Mapping with bwa-mem yielded similar results, but we chose Bowtie2 because we observed that it better distributes random reads between subtypes of the duplicates. The resulting total mapped reads were counted for each *SPATA31* copy and normalized by dividing the total number of mapped reads from the single copy reference house keeping genes. We then performed a linear regression analysis using normalized read depth (CNV) as the explanatory variable and the number of *de novo* mutations (obtained from GoNL (http://www.nlgenome.nl/?page_id=9)) as the response variable.

### DIGITAL PCR for copy number detection

We determined *SPATA31* copy numbers in two separate sample sets, in two independent runs. The first included DNA samples from 155 long-lived individuals (LLI - aged at least 96 years at the time of recruitment) and 163 samples of younger controls (60-75 years) from the same population cohort. The second included 96 samples from each of these groups. A detailed description of the study participants and the recruitment procedure is provided in [29] and [28]. Further, we used two different primer sets for each run, the first amplifying both, *SPATA31A* and *SPATA31C* variants, the second specific for *SPATA31A* variants.

All digital PCRs between control and LLI were run using the same master mix and same plate (e.g. 48 controls, 48 LLI for each run). To reduce the sampling error, genomic DNA was first diluted in 99μL by taking 1μL from 50ng/ μL stock concentration and mixed well by pipetting. Primer and probe sequences are provided in Suppl. Figure S8. In detail, the PCR reaction mixture was prepared from 12.5μL of 2x ddPCR Supermix for Probes (Bio-Rad, Hercules, CA, USA) mixed with 1.25μL of 20x primer-probe mix: 18μM PCR primers (each *SPATA31* primers, 5μM probe against SPATA31A mixed with 1.25μL of 20X primer-probe mix: 18μM PCR primers (each Albumin primers), 5μM probe against *SPATA31* and finally 0.1μL BamH1HF (NEB) added to the reaction. In total 15μL of reaction mixture was prepared in an Eppendorf 96-well twin.tec PCR plate. 10μL of diluted Genomic DNA (in total 5ng) was mixed with 15μL of the reaction mixture. The total 25μL reaction was mixed well by pipetting up and down and loaded into the Automated Droplet Generator (Bio-Rad, Hercules, CA, USA) to generate oil droplets in each well of the plate containing 20μL of the reaction mixture. After droplets were generated, the plate was sealed with a pierceable foil heat seal using PX1™ PCR Plate Sealer (Bio-Rad, Hercules, CA, USA) and then placed on a thermal cycler for amplification. Thermal cycling conditions were as follows: 95°C for 10 min (1 cycle), 94°C for 30 sec (ramp rate 2.5°C/sec) and 56°C for 60 sec (ramp rate 2.5°C/sec) (40 cycles), 98°C for 10 min (1 cycle), and 12°C hold. After PCR, the 96-well PCR plate was loaded on the QX100™ Droplet Reader (Bio-Rad, Hercules, CA, USA) which reads the droplets from each well of the plate. The data obtained were analyzed using QuantaSoft™ analysis software linked with the QX100™ Droplet Reader. We scored the copy numbers by measuring the concentration of the target, *SPATA31_All* or *SPATA31A* relative to the concentration of the reference *ALBUMIN*.

### Genome project analysis

We retrieved genome read data for 295 individuals (bam files) from the Simons Genome Project [26]. Reads were extracted and normalized as described above for the GoNL data.

### Data availability

RNASeq sequencing reads are available under SRA accession number PRJEB21178.

## Supplementary Material

Supplementary Figures

Supplementary Table S1

Supplementary Table S2

Supplementary Table S3

Supplementary Table S4

Supplementary Table S5
