# Supplementary material for: Involvement of SPATA31 copy number variable genes in human lifespan
Source: Aging (Albany NY). 2018 Apr 19;10(4):674–88. doi: 10.18632/aging.101421 (PMC5940121; doi:10.18632/aging.101421)
Supplement: Supplementary Figures [file aging-10-101421-s001.pdf]

SUPPLEMENTARY MATERIAL

Please browse the Full Text version to see Supplementary Tables related to this manuscript:

Supplementary Table S1. RNASeq results.

Supplementary Table S2. KEGG pathway analysis.

Supplementary Table S3. GoNL data analysis.

Supplementary Table S4. CNV data between control and LLI.

Supplementary Table S5. RNASeq statistics.

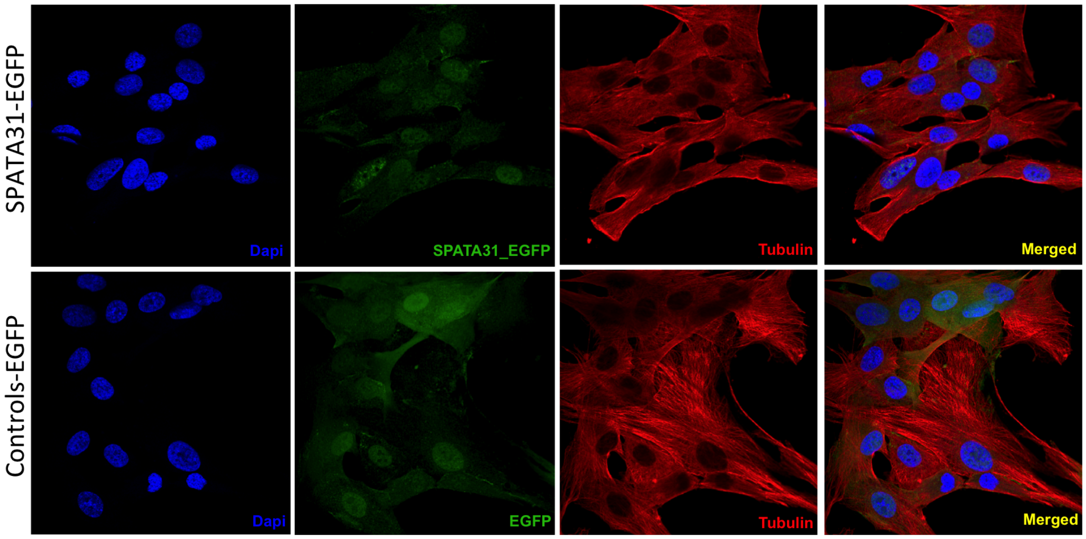

Supplementary Figure S1. Immunofluorescence of controls and SPATA31A1 over-expressing cells. Immunofluorescence analysis of control-EGFP and SPATA31A1-EGFP are presented. Control-EGFP and SPATA31-EGFP expressing cell lines for immuno-fluorescence analysis were grown in 24-well plates including previously added cover slips to each well. The growing media were removed and the cells were directly fixed with 0.5mL PBS/1.5% paraformaldehyde (PFA) for 10min at room temperature (RT) followed by -20°C cold methanol for 5 min at -20°C. Cells were washed three times with PBS and additionally washed with 1mL of PBS/0.1% saponin (Sigma-Aldrich) by incubating for 20min at RT on a shaker in slow motion (50 rpm). The wash buffer was removed and cells were immediately blocked by adding PBS/0.1% saponin/3% BSA (bovine serum albumin, fraction V, Sigma Aldrich) and incubated at RT in 24-well plates. Coverslips were further incubated with 0.25mL of PBS/0.1% saponin in a humified environment for 1h at RT including Mouse monoclonal α-tubulin antibody (Sigma 1:1000). Cells were washed 3x with 1mL of PBS/0.1% saponin. After washing, coverslips were incubated with the anti-mouse secondary antibody (Alexa Fluor® 546 (Molecular Probes, Life Technologies; GIBCO)) dilutions (1:2000) in a humidified environment for 1 hour at RT in the dark. Cells were washed 3x with 1mL of PBS/0.1% saponin for 20 min at RT on a shaker in slow motion (50 rpm). Finally, coverslips were put onto a microscope slide with 10µL of ProLong® Gold Antifade Mountant, which contains DAPI (Cat No: P36941, Molecular Probes, Life Technologies; GIBCO). After 2 hours of incubation at RT, cells were observed with a Leica (DM5000) confocal fluorescence microscope, using the Leica software (Leica Application Suite LAS X) for photography and analysis.

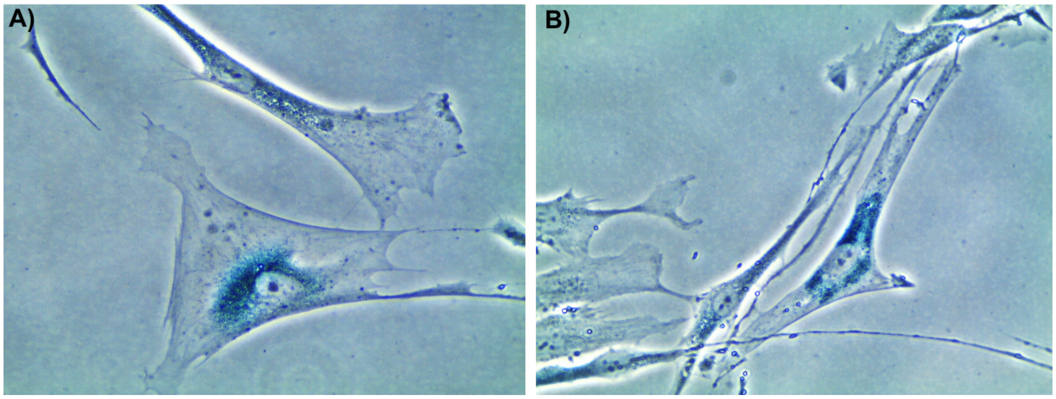

Supplementary Figure S2. ß-gal staining of controls and SPATA31A1 over expressing cells. Images show the example of staining results for senescence-associated ß-galactosidase for controls (**A**) and SPATA31 over expressing cells (**B**). Experiments were performed according to Kit instruction (Sigma B-gal senescence kit (Cat No:CS0030-1KT)). Please see methods for the detail of the analysis.

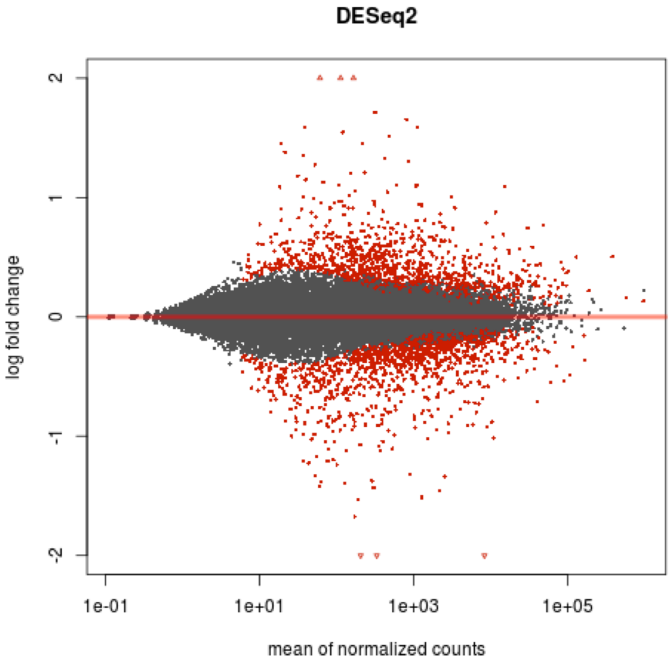

Supplementary Figure S3. MAplot of DeSeq2 analysis between control and treatment cultures. Red points represent values below 5% FDR.

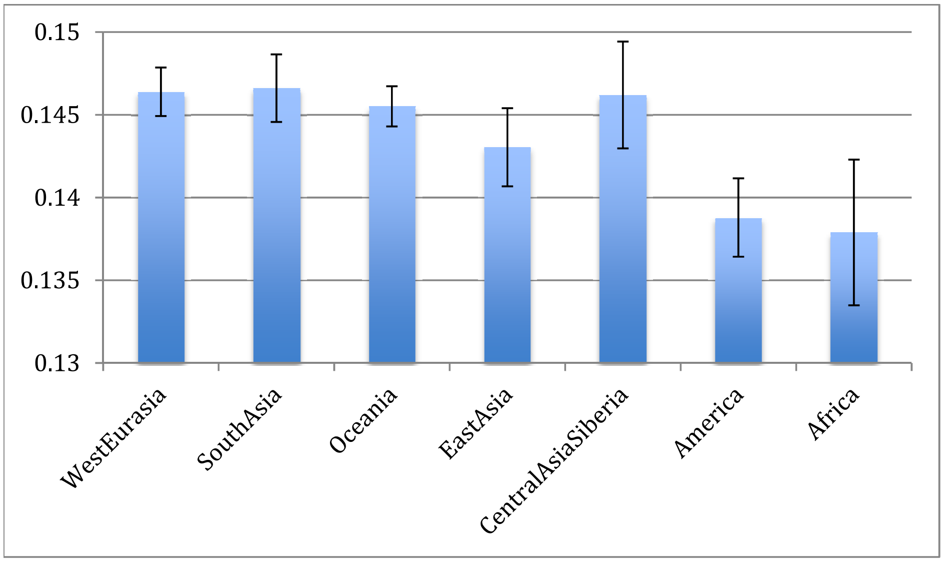

Supplementary Figure S4. Averages and variances of SPATA31 copy numbers in different human population groups. Data are based on normalized read depth from the Simons Genome Project data.

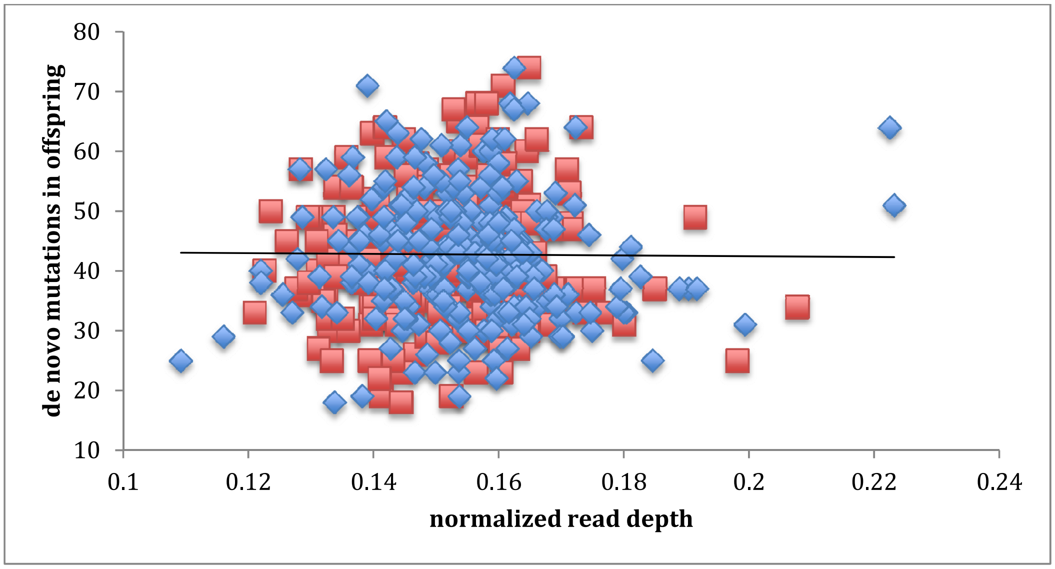

Supplementary Figure S5. Test for correlation between SPATA31 read depth in parents and number of de novo mutations in their offspring. Values for fathers (blue triangles) and mothers (red squares), the regression line is combined for both. There is no significant correlation for either or the combined dataset.

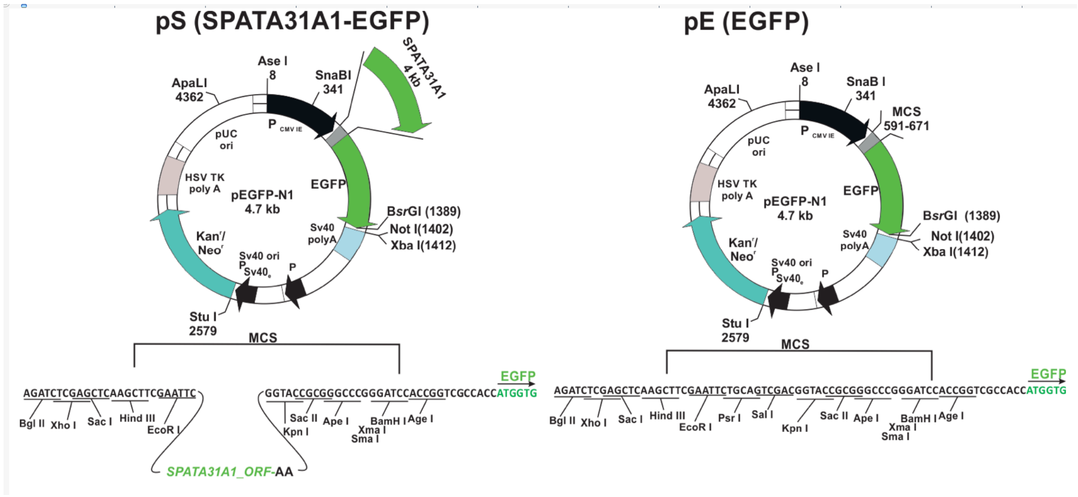

Supplementary Figure S6. Cloning and vector scheme. pS (SPATA31A1-EGFP) and pE (EGFP) constructs (modified after Adgene 6085-1, pEGFP-N1 vector).

Gene Name	Chromosome	Start	End
SPATA31A1	chr9	39355669	39361962
SPATA31A6	chr9	42183626	42189887
SPATA31A5	chr9	60914374	60920653
SPATA31A7	chr9	61190003	61196283
SPATA31A3	chr9	66986301	66992583
SPATA31P2	chr9	82057494	82063744
SPATA31C2	chr9	88129302	88135018
SPATA31C1	chr9	87917929	87923660
SPATA31P1	chr9	92882539	92888420
UBE2A	chrX	119574467	119584425
mTOR	chr1	11106535	11262507
TBP	chr6	170554333	170572869
B2M	chr15	44711477	44718877
TERT	chr5	1253167	1295047

Supplementary Figure S7. SPATA31A and reference gene locations and coordinates from hg38.

SPATA31_All

FM_All_F:	ACCACCTCAGTCTCCTCCCTAAGTG
FM_All_R:	GTGGGTGAGGGAAAAGTGCAGGT
FM_All_Probe:	5'FAM, TCCCAGCCACCAGAACCTTCCCT, 3'BHQ1
Amplicon Size:	100bp
Chromosomal Locations (hg38): Start-End	chr9:88132397-88132496<br>chr9:66989876-66989975<br>chr9:92885096-92885195<br>chr9:87920466-87920565<br>chr9:60916979-60917078<br>chr9:61192609-61192708<br>chr9:42186225-42186324<br>chr9:39358288-39358387

SPATA31_A_All

FM_A_R:	CTGTTTCGAGTTCTCTCCCATGTTC
FM_A_F:	ACAAGTCACAGAAACAGCCAAGGTC
FM_A_All_Probe:	5'FAM, ATCATTGGATGCTGAGCAGGACAC, 3'BHQ1
Amplicon Size:	184bp
Chromosomal Locations (hg38): Start-End	chr9:60917446-60917629<br>chr9:61193076-61193259<br>chr9:42186692-42186875<br>chr9:39358755-39358938<br>chr9:66989325-66989508

ALBUMIN

ALB_F:	TTGTGGGCTGTAATCATCGTCTAGG
ALB_R:	GCTGGTTCTCTTTCACTGACATCTGC
ALB_Probe:	5'HEX, CCCACACAAATCTCTCCCTGGCATT, 3'BHQ2
Amplicon Size:	115
Chromosomal Locations (hg38): Start-End	chr4:73418848-73418962

Supplementary Figure S8. Primers and Probes for the Digital PCR. Start and end position and fragment sizes for the Primers and Probes for listed. Please note that locations and fragment sizes for respective primer pairs were extracted from UCSC Genome Browser (hg38).
